# Construct Validity of the Sensory Profile Interoception Scale: Measuring Sensory Processing in Everyday Life

**DOI:** 10.3389/fpsyg.2022.872619

**Published:** 2022-05-13

**Authors:** Winnie Dunn, Catana Brown, Angela Breitmeyer, Ashley Salwei

**Affiliations:** ^1^Department of Occupational Therapy, College of Health Sciences, University of Missouri, Columbia, MO, United States; ^2^Department of Occupational Therapy, College of Health Sciences, Midwestern University, Glendale, AZ, United States; ^3^Department of Clinical Psychology, College of Health Sciences, Midwestern University, Glendale, AZ, United States

**Keywords:** interoception, measurement, construct validity, participation, sensory processing, occupational therapy, interoceptive impact

## Abstract

Scholars and providers are coming to realize that one’s ability to notice and respond to internal body sensations (i.e., interoception) contributes to an overall sense of wellbeing. Research has demonstrated a relationship between interoceptive awareness and anxiety, for example. Currently, however, tools for evaluating one’s interoception lack the conceptual foundation and clarity necessary to identify everyday behaviors that specifically reflect interoceptive awareness. Unlike existing interoceptive measures, the Sensory Profile Interoception (SPI) scale is participation-based and grounded in Dunn’s Sensory Processing framework. In this study we investigated concurrent validity by correlating the SPI with the Adolescent/Adult Sensory Profile (A/ASP); we investigated construct validity by correlating the SPI with the Perth Alexithymia Scale (PAS), the Body Awareness Scale (BAS), and the State-Trait Anxiety Inventory (STAI). Using the REDCAP online platform, 74 college-aged participants completed the measures. Using Spearman rank order correlations there were statistically significant relationships between the corresponding sensory pattern subscales on SPI and A/ASP (*r* = 0.277 to *r* = 0.582). The PAS was only weakly related to the registration subscale of the SPI (*r* = 0.260). The BAS had significant relationships with seeking and avoiding on the SPI (*r* = 0.496 and *r* = 0.385). The STAI had significant relationships with sensitivity and registrations of the SPI (*r* = 0.266 and *r* = 0.361 for state; *r* = 0.403 and *r* = 0.321 for trait). Taken together, these findings provide evidence of construct validity of the SPI to identify participation patterns associated with both high and low interoception. With the more precise information the SPI provides, professionals can design tailored interventions to support everyday life goals and researchers can study interoception within authentic activities.

## Introduction

There are many bodily functions that we take for granted because they operate outside of our momentary awareness unless something is awry. Although neuroscientists have studied the internal body sensations systems (i.e., interoception) many decades ago (Kandel et al., 2013; Ceuhen et al., 2016), applied scientists have only more recently paid attention to the relationship between one’s awareness of internal body sensations and various conditions like autism, anxiety, hypochondria, suicide attempts, and anorexia, to name a few (Engel-Yeger et al., 2013; Forrest et al., 2015; Longarzo et al., 2015; Critchley and Garfinkel, 2017; Fiene et al., 2018; Khoury et al., 2018; Abdulhamid et al., 2021).

An understanding of interoception and related conditions provides insight into how both interoceptive awareness and unawareness manifests as symptoms. Many studies have established that alexithymia (the inability to identify and describe one’s emotions) is associated with lower levels of interoception, and there is also evidence that atypical interoceptive perception might result in compromised ability to regulate body functions such as breathing (Abdulhamid et al., 2021). Similarly, adults with autism have high rates of interoceptive confusion (74%), and this lack of awareness of bodily states is associated with alexithymia (Fiene et al., 2018). Anxiety is naturally associated with interoception as many of the symptoms include bodily reactions such as shortness of breath and racing heartbeat. One model suggests that people with anxiety have poor predictive ability at interpreting interoceptive input to determine if it signals a true threat (Paulus and Stein, 2010). This poor predictive ability brings about a constant state of uncertainty for the future. A review of interoception and anxiety highlights the role that body awareness plays in learning and the exertion of a behavioral response (Van Diest, 2019). In panic disorder, interoceptive awareness in the form of feelings of breathlessness results in a conditioned response of cardiorespiratory fear and arousal. In yet another example, eating disorders are associated with a mistrust of bodily sensations (Martini et al., 2021).

Existing interoceptive measures use both physiological and self-report approaches. Garfinkel et al. (2015) suggest there are three dimensions of interoception that must be distinguished. First “interoceptive accuracy” refers to one’s ability to report about internal body sensations when compared to physiological measures. Secondly, “interoceptive sensibility” includes one’s self-perceived ability to notice internally body sensations. Finally, “interoceptive awareness” describes one’s metacognitive ability to recognize the synchrony of one’s reporting with physiological readings of internal sensations. For example, physiological measures, such as heartbeat tasks, quantify how accurately a person can detect their own heartbeat at rest (Schandry, 1981). Other measures use self-report as a means of capturing an individual’s self-perception of acuity or attention to interoception. Some self-report interoception measures focus on physiological observations (e.g., “I have an extra strong heartbeat” I feel when my bowels contract; e.g., Longarzo et al., 2015; Cabrera et al., 2018; Vlemincx et al., 2021). Others link attention to interoception as part of interoceptive sensibility (e.g., “When I’m short of breath, I focus on this”; Bogaerts et al., 2021; “I can return awareness to my body if I am distracted,” Mehling et al., 2018). Desmedt et al. (2022) examined current interoception questionnaires to examine whether they were testing a common interoceptive construct. They found that the most frequently used assessments each tested a different aspect of interoceptive sensibility. They call for additional work on both conceptualization of interoception and on clearly articulating these conceptualizations within assessments.

Yet, interoception has an impact on the person that goes beyond physiological responses, awareness of internal sensations, emotions, cognition, and symptoms. The experience of bodily sensations influences participation in daily life. The International Classification of Functioning, Disability and Health (ICF) (World Health Organization [WHO], 2001) includes physiological responses and internal body awareness as “body functions and structures.” The ICF also includes “participation” as a key factor in health; the ICF defines participation as “involvement in life situations.” For example, if someone is feeling breathless at work, they may choose to step outside for some fresh air. If someone lacks an awareness of the need to empty their bladder, they may experience accidents that could affect the ways they socialize with others. A person who notices the effect of caffeine on their body, may choose to avoid coffee later in the day so that they can get to sleep at a reasonable time. For a comprehensive conceptualization to evolve, researchers must also consider the influence of interoception on everyday life (i.e., “interoceptive impact”). Perhaps this current study extends Garfinkel et al.’s (2015) model to include this fourth dimension.

A greater appreciation for the role of interoception and symptoms has led to the development of interventions targeting interoception. There is evidence suggesting that mindfulness-based interventions can lead to increased noticing of interoception input, resulting in decreased stress and increased wellbeing (Fazia et al., 2021). In another study, women in treatment for substance use disorder experienced improvements in interoceptive awareness, emotion regulation and days abstinent after participating in a mindfulness intervention centered on body-awareness (Price et al., 2019). A systematic review of cognitive-behavior therapy for panic disorder found that the most effective interventions were those that were administered face-to-face and included interoceptive exposure (Pompoli et al., 2018). However, many practitioners are also interested in interventions with outcomes related to activities of daily living, instrumental activities of daily living, work, leisure, and socialization. Targeting participation as an outcome is best when the assessment addresses the link between interoception and participation. For example, a participation-based interoceptive measure may alert the practitioner to potential leisure restrictions because the individual is concerned with getting hurt.

When using an interoception measure for intervention planning with outcomes focused on participation, it is important that the measure link internal awareness to activities people do in their daily lives. For example, if a person fails to notice hunger or thirst (low registration of interoceptive cues) which interferes with concentration, we can collaborate to design reminders throughout the day.

The Adolescent/Adult Sensory Profile (A/ASP; Brown and Dunn, 2002) is widely accepted as an important measure for assessing sensory processing in daily life. The assessment is used for both research and clinical practice (e.g., Pfeiffer et al., 2005; Rieke and Anderson, 2009; Chung and Song, 2016; Howe and Stagg, 2016; Bijlenga et al., 2017). The A/ASP is built on an evidence-based conceptual framework (Dunn, 2014) to characterize four sensory processing preferences: sensitivity, avoiding, registration, and seeking. When the A/ASP was developed, seven senses were included: seeing, hearing, tasting, smelling, touching, moving (vestibular), and posturing (proprioception). The availability of an interoceptive measure based on Dunn (2014) provides a conceptual structure for intervention planning. Providers can obtain more details about interoceptive awareness from the four sensory processing preferences (seeking, avoiding, sensitivity, registration) to improve participation. Those who seek interoceptive input require interventions which facilitate more interoceptive activity (e.g., increasing the strenuousness of an activity), while those who are sensitive to interoceptive input require individualized plans that limit interoceptive input (e.g., planning controlled episodes of strenuousness).

In pilot studies focused on item development, researchers clarified wording, evaluated consistency with a conceptual framework (Dunn’s Sensory Processing Framework) using item to scale correlations, internal consistency tests (Chronbach’s alpha) and scale to scale correlations [A/ASP to Sensory Profile Interoception (SPI)], and edited the items based on feedback (Brown and Dunn, 2020). The current version of the SPI will benefit from further psychometric analysis that considers the relationship of the SPI with measures that capture related information already associated with interoception (anxiety, alexithymia, body awareness). If relationships are identified, this would support the construct validity of the SPI. Similarly, construct validity can be examined by determining the relationship between the SPI and Dunn (2014); the A/ASP contains subscales for the sensory patterns in DSPF, i.e., seeking, avoiding, sensitivity, and registration.

The purpose of this study was to evaluate the concurrent (A/ASP) and construct validity (other measures) of the Sensory Profile Interoception (SPI) scale, a participation-focused measure of internal body sensations. We expected that there would be positive correlations between low threshold sensory patterns (Sensitivity and Avoiding) and measures of body awareness and anxiety based on prior literature about interoception (Forrest et al., 2015; Longarzo et al., 2015; Critchley and Garfinkel, 2017; Fiene et al., 2018; Khoury et al., 2018; Abdulhamid et al., 2021). Similar to other researchers, we expected that alexithymia would be related to registration since both of these constructs reflect a failure to detect stimuli (Dunn, 2014; Fiene et al., 2018), and we hypothesized that the SPI sensory patterns would correlate most strongly with the corresponding sensory patterns of the A/ASP (Brown and Dunn, 2002) since we built items for the SPI using the same conceptual framework (DSPF; Dunn, 2014). Additionally, the SPI has the potential to expand the interoceptive framework of Desmedt et al. (2022) to include “interoceptive impact” based on the ICF model (World Health Organization [WHO], 2001) of function, disability and health.

## Materials and Methods

We conducted a correlational analysis to evaluate the construct validity of the SPI.

### Participants

We recruited a convenience sample of students from occupational therapy programs at three universities and a behavioral science program at one of these universities. We sent emails to students inviting them to participate and provided a link to the secured server. We accepted all students who chose to participate because we believed this sample would have an adequate range of scores on the various measures.

### Procedures

After obtaining IRB approval from each university and permission from chairs of each department, we sent emails to the students asking them to participate. The email included a description of what they would be doing and a link to a REDCAP survey, which held all the demographic and test items. We told participants that completing the REDCAP questions would serve as their permission to participate. Every participant received a code to track their answers; no personally identifiable information was collected.

### Measures

#### Sensory Profile Interoception

The SPI is a newly developed scale to evaluate how interoception manifests itself in everyday life behaviors, particularly related to self-care (activities of daily living, ADL) (*n* = 29 items), eating (*n* = 25 items) and daily routines (instrumental activities of daily living, IADL) (*n* = 37 items). We designed the SPI for adolescents, young and older adults to align with the Adolescent/Adult Sensory Profile (A/ASP).

In pilot studies focused on item development, researchers clarified wording, evaluated consistency with a conceptual framework (Dunn, 2014), and edited the items based on feedback (Brown and Dunn, 2020). In the first study, we identified items from the literature, wrote items using the A/ASP as a guide to sensory pattern language, and solicited items from colleagues. We held focus groups with colleagues to obtain their feedback about clarity of items. Then we edited items and recruited a convenience sample of adults to take the SPI. We revised items to improve internal consistency. We used findings from this second pilot study to revise the SPI so that its structure reflects DSPF.

There are currently 91 items on the SPI, and respondents indicate the frequency with which they engage in the behaviors described in each item on a 5-point Likert scale (1 = almost never to 5 = almost always). The SPI is divided into four subscales, that mirror and are theoretically consistent with the sensory patterns of the Adolescent/Adult Sensory Profile described below. The sensitivity subscale (n-30 items) indicates a heightened awareness of interoception, the avoiding subscale (*n* = 25 items) is an assessment of active behaviors to avoid interoceptive sensations, the (low) registration subscale (*n* = 20 items) reveals a lack of awareness of interoceptive input, and the seeking subscale (*n* = 16 items) signals active behaviors to increase interoceptive input. These items have been tested in pilot studies (Brown and Dunn, 2020) to: a. clarify wording of items, and b. to evaluate consistency with DSPF using the Adolescent/Adult Sensory Profile. In the prior study we used item to subscale correlations, Chronbach’s alpha within subscales to test for consistency and correlations between the SPI and the A/ASP to determine which items were strongest. There was good internal consistency (α = 0.63–0.88) and significant/moderate correlations with sensory patterns on the A/ASP (Seeking *r* = 0.311, *p* = 0.032; Registration *r* = 0.378, *p* = 0.009; Sensitive *r* = 0.448, *p* = 0.002, Avoiding *r* = 0.323, *p* = 0.031), suggesting both convergent and divergent validity of the scale. Table 1 provides examples of the SPI items.

**TABLE 1:** Examples of items from the Sensory Profile Interoception scale.

	Eating	Activities of daily living	Instrumental activities of daily living
Seeking	“I eat whatever I want whenever I want.”	“I like lots of blankets for sleeping.”	“I enjoy activities that make my heartbeat faster (e.g., vigorous physical activity, amusement park rides, scary movies).”
Avoiding	“I stay away from new foods because I do not know how they will make me feel.”	“Sleeping is elusive to me.”	“I stay away from activities where I think I could get hurt.”
Sensitivity	“I am careful about what I eat because only certain foods settle in my stomach.”	“I have particular brands of hygiene products that are OK.”	“My ears/face get hot during stressful meetings.”
Registration	“I realize I need to drink something after it is too late (e.g., feel lightheaded, extremely thirsty).”	“I find a razor cut later in the day.”	“I get bruises or other injuries and I do not remember how the injury happened.”

#### Adolescent/Adult Sensory Profile

The A/ASP (Brown and Dunn, 2002) is widely accepted within and outside of occupational therapy as an important participation-based measure for assessing sensory processing. The A/ASP uses Dunn’s (2014) Sensory Processing Framework to characterize four sensory processing preferences: sensitivity, avoiding, registration, and seeking. In addition, the measure includes items representing seven different sensory modalities: vision, hearing, taste, smell, touch, vestibular (body movement), and proprioception (body position). Respondents record how frequently they engage in the behaviors described on 60 items using a 5-point Likert scale (1 = almost never to 5 = almost always). The A/ASP has good internal consistency (0.66–0.82), and validity has been established in the literature over the past two decades (e.g., Pfeiffer et al., 2005; Rieke and Anderson, 2009; Chung and Song, 2016; Gonthier et al., 2016; Howe and Stagg, 2016; Bijlenga et al., 2017; Mayer, 2017; Gándara-Gafo et al., 2019; Zaree et al., 2021).

#### Perth Alexithymia Questionnaire

The Perth Alexithymia Questionnaire measures difficulty attending to and assessing one’s own feelings. This scale was chosen because many studies find alexithymia is associated with poor interoception (Abdulhamid et al., 2021). The PAQ is a 24-item questionnaire, and respondents mark on a 7-point scale (1 = not at all true of me to 7 = very true of me). The scale is divided into 5 scales: (1) negative and (2) positive difficulty identifying feelings, (3) negative, and (4) positive difficulty describing feelings, and (5) a general external oriented thinking scale. There is support for the measure’s concurrent and discriminant validity and internal consistency, i.e., α ≥ 0.80 for all PAQ subscales (Preece et al., 2018, 2020), and it has an advantage over some other measures in that it assesses both positive and negative emotions (Preece et al., 2020).

#### State/Trait Anxiety Inventory

The State-Trait Anxiety Inventory for adults is a well-established and widely used assessment of anxiety symptoms (STAI; Spielberger et al., 1983). This scale was chosen because anxiety is considered by many to include a heightened sensitivity to interoception (Mallorquí-Bagué et al., 2016). There are 20 items related to anxiety and 20 items related to general state. Respondents mark on a 7-point scale (1 = strongly disagree, 4 = neither agree nor disagree, 7 = strongly agree). The measure has excellent test-retest reliability (average *r* = 0.88), excellent internal consistency (average α > 0.89), and good discriminate validity (Metzger, 1976; Barnes et al., 2002).

#### Body Awareness Questionnaire

The Body Awareness Questionnaire (Shields et al., 1989) is an assessment of interoception that does not include emotional body processes. Although the scale is not divided into subscales, some of the items are more reflective of awareness of bodily reactions (consistent with sensitivity in the interoception scale), while others address the impact of habit or routine on body functions (consistent with avoiding on the interoception scale). There are 18 items, and respondents rate them on 7-point scale (1 = not at all true of me to 7 = very true of me). There is support for the measure’s convergent and discriminant validity (Shields et al., 1989), test-retest reliability (*r* = 0.80), internal consistency (α range of 0.77–0.83; Shields et al., 1989), and concurrent validity (Unal et al., 2020).

### Data Analysis

We conducted a set of descriptive analyses to describe our participants. We used correlations to examine the relationships among the SPI and other benchmark measures. Specifically, we completed Spearman Rank Order Correlations between summary scores on the SPI and summary scores on the other measures. We used Spearman Rank Order Correlations because this calculation does not assume linearity.

## Results

We describe our results based on our hypotheses. Seventy-four students participated in the study. Eighty-five percent (*n* = 63) were White, 1.5% (*n* = 1) was black or African American, 4% (*n* = 3) were Hispanic or Latinx, 4% (*n* = 3) were Pacific Islanders, and 5.4% (*n* = 4) reported “other”; 90% were female (*n* = 67). They ranged in age from 21 to 45 years, with a mean of 26 years old and a standard deviation of 4.6 years. Eleven participants were 30 years of age or older. Table 2 provides the means and standard deviations for the other measures in the study. There were no differences between age groups (21–23 years, *n* = 22, 24–29 years, *n* = 41, 30 years and older, *n* = 11) on the measures in the study based on an Analysis of Variance by age (Hotelling’s Trace = 0.752, *F* = 1.128, significance = 0.338). We did not collect any additional information about the participant demographics.

**TABLE 2:** Means and standard deviations of the measures in the study.

	Mean	SD
State	38.70	11.39
Trait	43.38	10.06
Iavoid	66.89	8.41
Isens	83.20	13.27
Ireg	37.77	8.94
Iseek	40.56	8.61
ALEX	70.90	33.30
BAQ	80.90	15.32

*State, State Anxiety Scale; Trait, Trait Anxiety Scale; Iavoid, Avoiding subscale of the SPI; Isens, Sensitivity subscale of the SPI; Ireg, Registration subscale of the SPI; Iseek, Seeking subscale of the SPI; ALEX, Alexithymia Scale total; BAQ, Body Awareness Questionnaire total.*

Table 3 provides the correlations between the subscales of the A/ASP and the SPI. Supplementary Appendix 1 contains scatterplots of significant correlations. The constructs of Dunn’s Sensory Processing Framework (DSPF) were correlated across the A/ASP and the SPI. All of the SPI subscales correlated most strongly with their corresponding A/ASP subscale except for the avoiding subscale, with its highest correlation with the A/ASP sensitivity subscale (*r* = 0.366), but only a slightly lower correlation with the A/ASP avoiding subscale (*r* = 0.338). These findings support the SPI’s concurrent validity in terms of the measure’s consistency with its theoretical basis (the DSPF) (see Table 1 for examples), and a participation-based sensory processing measure. As outlined in Table 3, seeking is only correlated with the corresponding subscale between the 2 scales (seeking = 0.523). Registration and sensitivity have their highest correlations with their corresponding subscales (registration = 0.552; sensitivity = 0.582), although these scales also have significant correlations with other subscales.

**TABLE 3:** Correlations between A/ASP and SPI subscales (*n*-74).

	SPI registration	SPI seeking	SPI sensitivity	SPI avoiding
A/ASP registration	0.552**	0.113	0.447**	0.009
A/ASP seeking	0.392**	0.523**	0.339**	0.187
A/ASP sensitivity	0.423**	0.093	0.582**	0.366**
A/ASP avoiding	0.277*	0.091	0.373**	0.338**

*p < 0.05, **p < 0.01 two tailed.

*Significant correlations are plotted in Supplementary Appendix 1. A/ASP, Adolescent/Adult Sensory Profile; SPI, Sensory Profile Interoception.*

Consistent with our hypotheses, we found that alexithymia had a small but significant correlation with the registration score on the SPI. Body awareness had moderate correlations with avoiding and seeking and a small but significant correlation with the sensitivity scores on the SPI. The State/Trait Anxiety measure had moderate correlations with sensitivity and registration on the SPI. Table 4 provides the correlations. Supplementary Appendix 1 provides scatterplots of significant correlations.

**TABLE 4:** Correlations between alexithymia, body awareness, and anxiety to the Sensory Profile Interoception scale (*n* = 74).

	SPI Avoiding	SPI Sensitivity	SPI registration	SPI seeking
Perth alexithymia scale	−0.047	0.138	0.260*	−0.000
Body awareness questionnaire	0.385**	0.256*	0.002	0.496**
State anxiety	0.143	0.266*	0.361**	−0.016
Trait anxiety	0.142	0.403**	0.321**	−0.035

*p < 0.05, **p < 0.01 two tailed.

*SPI, Sensory Profile Interoception; PAS, Perth Alexithymia Scale; BAS, Body Awareness Scale; State and Trait are parts of the State/Trait Anxiety Scale. Significant correlations are plotted in Supplementary Appendix 1.*

## Discussion

In this study, we examined the validity of the newly developed SPI in two ways. First, we examined the relationship between the SPI and the A/ASP to determine whether the SPI has a strong conceptual foundation, specifically Dunn’s Sensory Processing Framework (DSPF), with an established participation-based measure of sensory processing (i.e., the A/ASP) (concurrent validity). Secondly, we examined the relationship between the SPI and related mental health measures to determine whether the SPI reflects known relationships between interoception and psychological factors (construct validity). We provide examples of the SPI items in Table 1.

Knowing more precisely how interoceptive awareness will affect every day routines provides knowledge for effective intervention planning at the participation level. The State Anxiety scale includes “I feel tense” and “I feel upset”; the Trait Anxiety scale includes “I feel nervous and restless.” The SPI provides more detail about how “tense,” “nervous,” and “upset” might affect one’s daily routine, such as “I worry about stomach/digestive processes” and “I select hobbies that are orderly and predictable so I can stay calm” which indicate possible focus for intervention planning (e.g., in these examples, eating, or hobbies). Additionally, by also reflecting the four sensory patterns from DSPF, more precision is possible. For example, “I stay away from activities where I think I could get hurt” (avoiding), “I get bruises or other injuries, and I do not remember how the injury happened” (registration), “I enjoy activities that make my heart beat faster (e.g., vigorous physical activity, amusement park rides, scary movies)” (seeking), and “I try to control my heartbeat when it becomes too fast (e.g., by slowing down, meditating)” (sensitivity) suggest different approaches to supporting participation.

### Concurrent Validity With the Conceptual Foundation and Participation-Focused Framework of the Sensory Profile Interoception

The SPI seems to reflect DSPF. All corresponding sensory pattern subscales (i.e., seeking, avoiding, sensitivity, registration) had significant correlations between the SPI and the A/ASP, and, in all cases but avoiding, these subscales correlated most strongly with their corresponding subscale (for example, the A/ASP seeking subscale and the SPI seeking subscale). As an illustration, “I find a razor cut later in the day” from the SPI is significantly correlated with several low registration items on the A/ASP: “I trip or bump into things,” “I am unsure of footing when walking on stairs,” and “I miss the street, building or room signs when trying to go somewhere new,” while being unrelated to A/ASP items reflecting other sensory patterns. Additionally, the correlations were moderate, suggesting the SPI is assessing some additional information not covered by the A/ASP. The SPI seems to reflect the strong conceptual base of DSPF seen across many studies of the Child and Adult Sensory Profiles (e.g., Little et al., 2018; Nesayan et al., 2018; Tomchek et al., 2018; Zaree et al., 2021). This foundation gives providers and researchers a clear structure for discussing findings about interoception and linking to other systems that might be relevant to a specific research question or intervention planning.

The seeking subscales only correlated with each other, suggesting that seeking has some distinct characteristics that are present on both the SPI and the A/ASP. As reported in prior literature, the low threshold sensory patterns (avoiding and sensitivity) correlate with their corresponding pattern (i.e., avoiding with avoiding, sensitivity with sensitivity), and with each other. This trend is also consistent with prior literature, which suggests there is a continuum of high responsiveness to sensory input (low sensory thresholds). Finally, registration, which reveals missing cues in the environment, is most highly correlated with its counterpart as well.

### Construct Validity of Sensory Profile Interoception as Related to Psychological Factors

We examined construct validity with psychological factors by correlating the SPI with alexithymia, body awareness and anxiety measures. The SPI differs from other interoception measures because the quadrants allow for consideration of level of awareness of interoception (sensitivity and registration) as well as active efforts to regulate interoceptive input (avoiding and seeking quadrants).

As anticipated, the Perth Alexithymia Scale was correlated with the registration score on the SPI, which was the only significant correlation. Alexithymia is an inability to detect and describe one’s emotions; registration scores (as it is tested on the SPI and the A/ASP) indicate the amount that a person misses cues in the environment. This finding is consistent with other studies indicating a relationship between poor interoception and alexithymia (e.g., Abdulhamid et al., 2021).

The Body Awareness Questionnaire related to all the subscales except registration. As expected, the Body Awareness Questionnaire was associated with the SPI sensitivity scale, which assesses interoceptive awareness. However, the highest correlations were with the seeking and avoiding scales. Both seeking and avoiding are measuring active self-regulation patterns; perhaps active self-regulation patterns contribute to knowing about one’s body. Since registration on the SPI evaluates how frequently people miss cues in their everyday lives, it is not surprising that with our sample from a general population, registration on the SPI would not be correlated to body awareness as tested on the BAQ.

State and trait anxiety were related to sensitivity and registration. Patterns of both noticing and missing input is consistent with other studies examining anxiety and sensory processing. In the second edition of the Toddler Sensory Profile (Dunn, 2014) and in other studies with adults (Engel-Yeger and Dunn, 2011; Engel-Yeger et al., 2016, 2018; Brown et al., 2020), there is a small but significant relationship between registration and conditions such as anxiety, hypochondria, anorexia, and pain catastrophizing (Forrest et al., 2015; Longarzo et al., 2015; Critchley and Garfinkel, 2017; Fiene et al., 2018; Khoury et al., 2018; Abdulhamid et al., 2021). We believe that people with registration tendencies do miss cues as we originally believed. However, at some point, bystanders (people with low registration) do notice a possible catastrophe, but it is so late in the situation that a big response is necessary (Little et al., 2016). This big response looks remarkably like a sensor’s responses; it is the timing that is different. In addition, Clark et al. (2018) found high sensitivity and registration from the A/ASP and high trait anxiety in people with chronic low back pain. However, they also found high sensation avoiding. They hypothesize that premorbid trait anxiety and sensory processing patterns contribute to pain experiences.

It is unclear as to why avoiding on the SPI was not correlated with trait or state anxiety, but it may be that this relationship is more detectable in a clinical population. Alternatively, the regulation through avoiding may be different for interoceptive input. People with avoiding tendencies are more likely to anticipate difficult situations and stop them, for instance not attending a party or turning down an invitation to a public event. However, with the SPI, avoiding is characterized as an active behavioral response that can be adaptive in terms of managing unpleasant sensations. Anxiety is associated with passive coping strategies, which can include a general lack of engagement in life (LeDoux and Gorman, 2001). People with sensitivity try to participate and then find themselves overwhelmed, which is consistent with anxiety laden behaviors and thoughts (Dunn et al., 2016a,b).

### Limitations of This Study

We recruited a convenience sample of students for this study, which introduces sampling bias. It is possible that students in occupational therapy and behavioral sciences would be more aware of interoception and behavior, although participants had not completed their professional education at the time of participation. We also had a majority of white female participants, which could also bias our outcomes. Future studies must expand the demographics to learn how these data fit into the bigger picture.

## Conclusion

The concurrent validity of the four-quadrant model of the SPI was supported by the correlation of the SPI with the Adolescent/Adult Sensory Profile. The low to moderate correlations with the psychological factors (construct validity) already related to interoception suggest that the SPI reflects related concepts while being a distinct assessment of interoception. As the first participation-based interoception assessment, the SPI may be particularly useful for clinical use when intervention goals focus on participation-oriented outcomes.

## Data Availability Statement

The datasets presented in this article are not readily available because the measure described by the authors is under development and we would want to approve of any use of the data before offering it to others. Requests to access the datasets should be directed to cbrown2@midwestern.edu.

## Ethics Statement

The studies involving human participants were reviewed and approved by Midwestern University Institutional Review Board. Written informed consent for participation was not required for this study in accordance with the national legislation and the institutional requirements.

## Author Contributions

WD and CB contributed to the conceptualization and design of the study, data analysis, and writing and editing of the manuscript. AB contributed by assisting with the selection of relevant assessment and editing of the manuscript. AS contributed by assisting with data collection and editing of the manuscript. All authors contributed to the article and approved the submitted version.

## Conflict of Interest

WD and CB were authors of the Adolescent/Adult Sensory Profile, a measure that was used in this study. We receive royalties for the assessment. The remaining authors declare that the research was conducted in the absence of any commercial or financial relationships that could be construed as a potential conflict of interest.

## Publisher’s Note

All claims expressed in this article are solely those of the authors and do not necessarily represent those of their affiliated organizations, or those of the publisher, the editors and the reviewers. Any product that may be evaluated in this article, or claim that may be made by its manufacturer, is not guaranteed or endorsed by the publisher.
