# Corrigendum: Construct Validity of the Sensory Profile Interoception Scale: Measuring Sensory Processing in Everyday Life

**DOI:** 10.3389/fpsyg.2022.948352

**Published:** 2022-08-03

**Authors:** Winnie Dunn, Catana Brown, Angela Breitmeyer, Ashley Salwei

**Affiliations:** ^1^Department of Occupational Therapy, College of Health Sciences, University of Missouri, Columbia, MO, United States; ^2^Department of Occupational Therapy, College of Health Sciences, Midwestern University, Glendale, AZ, United States; ^3^Department of Clinical Psychology, College of Health Sciences, Midwestern University, Glendale, AZ, United States

**Keywords:** interoception, measurement, construct validity, participation, sensory processing, occupational therapy, interoceptive impact

In the original article, in the reference Fazia et al. (2021) one of the author's names was incorrectly spelled. The reference was written as: “Fazio, T., Bubbico, F., Berzuini, G., Tezza, L. D., Cortellini, C., Bruno, S., and Bernardinelli, L. (2021). Mindfulness meditation training in an occupational setting: effects of a 12-weeks mindfulness-based intervention on wellbeing. Work 70, 1089–1099. doi: 10.3233/WOR-210510” but it should be “Fazia, T., Bubbico, F., Berzuini, G., Tezza, L. D., Cortellini, C., Bruno, S., and Bernardinelli, L. (2021). Mindfulness meditation training in an occupational setting: effects of a 12-weeks mindfulness-based intervention on wellbeing. Work 70, 1089–1099. doi: 10.3233/WOR-210510”.

The authors apologize for this error and state that this does not change the scientific conclusions of the article in any way. The original article has been updated.

## Publisher's Note

All claims expressed in this article are solely those of the authors and do not necessarily represent those of their affiliated organizations, or those of the publisher, the editors and the reviewers. Any product that may be evaluated in this article, or claim that may be made by its manufacturer, is not guaranteed or endorsed by the publisher.
