# Supplementary material for: Construct Validity of the Sensory Profile Interoception Scale: Measuring Sensory Processing in Everyday Life
Source: Front Psychol. 2022 May 13;13:872619. doi: 10.3389/fpsyg.2022.872619 (PMC9137433; doi:10.3389/fpsyg.2022.872619)

**APPENDIX 1: SCATTERPLOTS OF SIGNIFICANT CORRELATIONS**

**SCATTERPLOTS for Table 3 significant correlations**

SPI registration with A/ASP registration

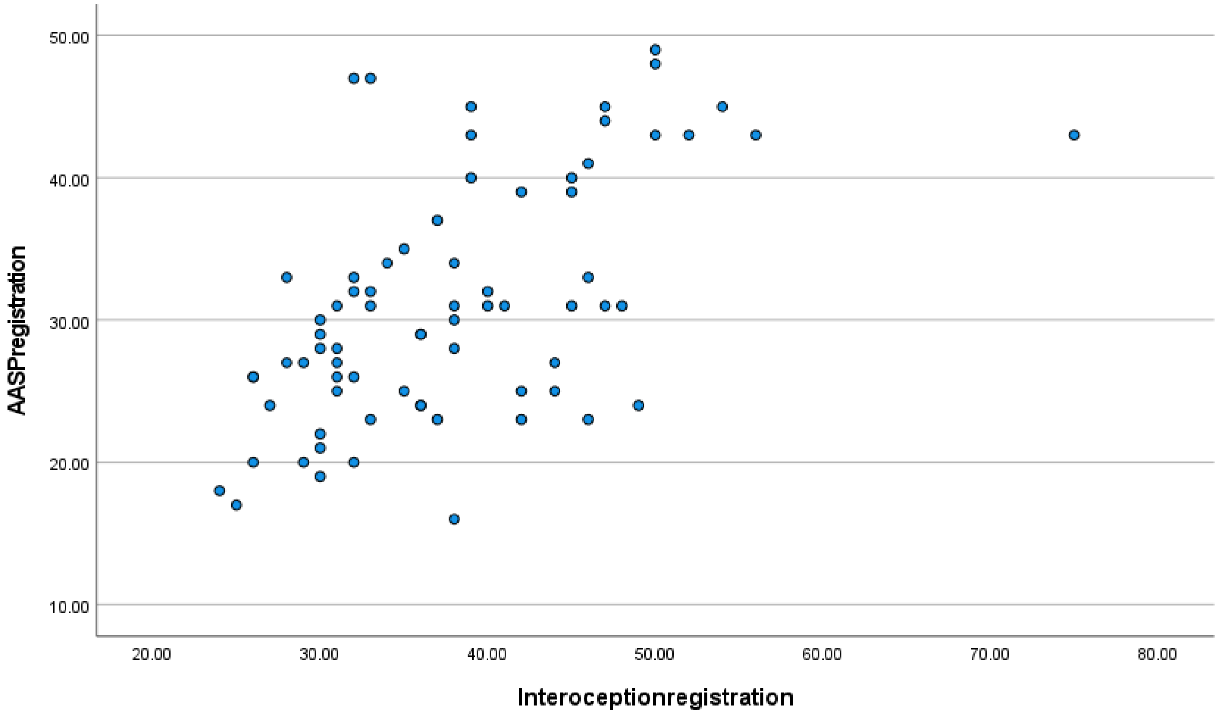


SPI registration with A/ASP seeking

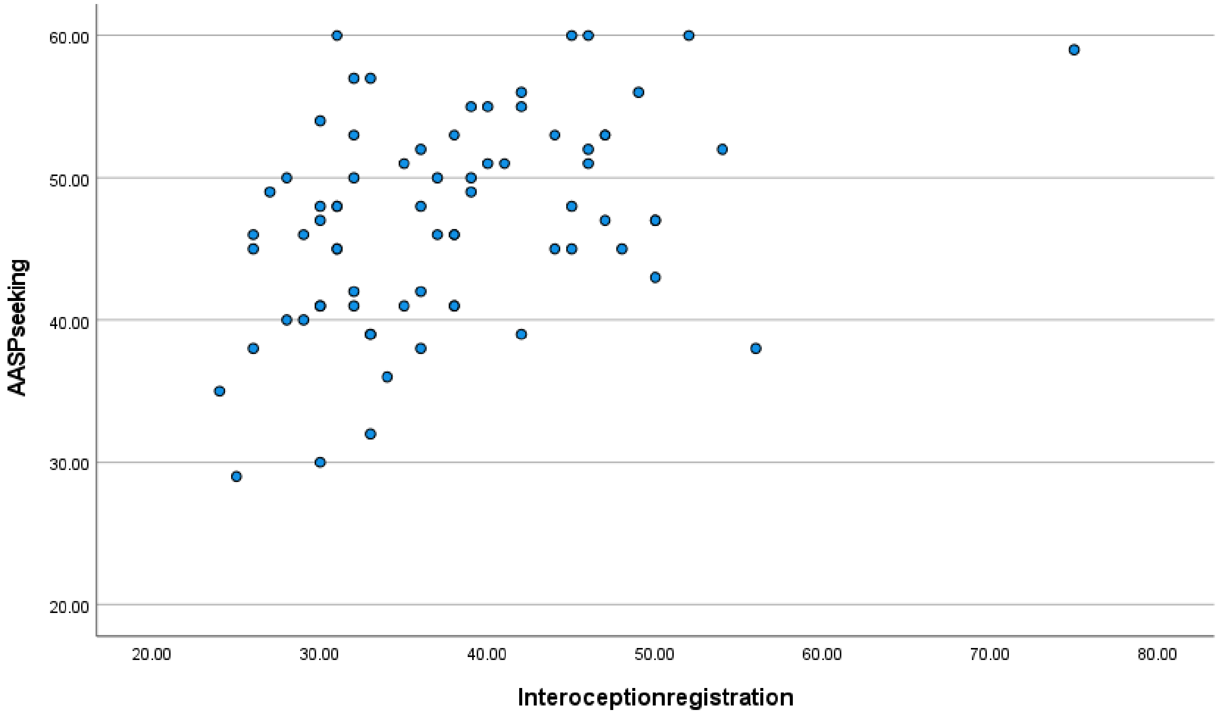

SPI registration with A/ASP sensitivity

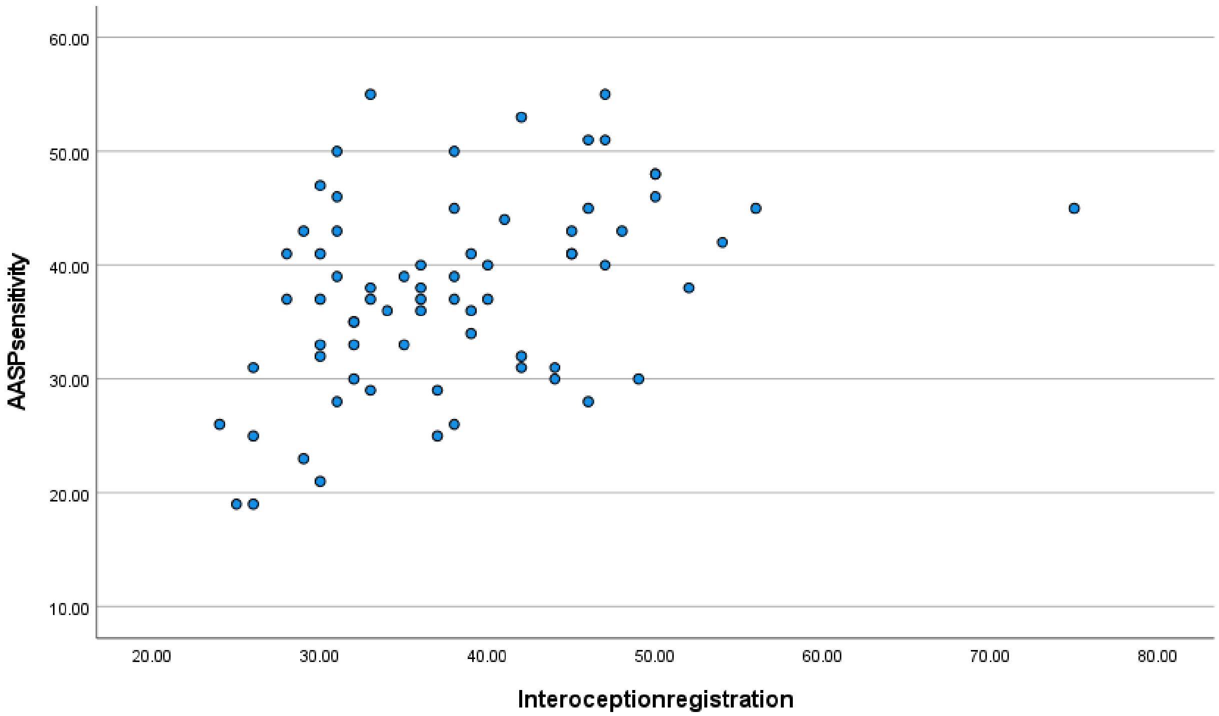


SPI registration with A/ASP avoiding

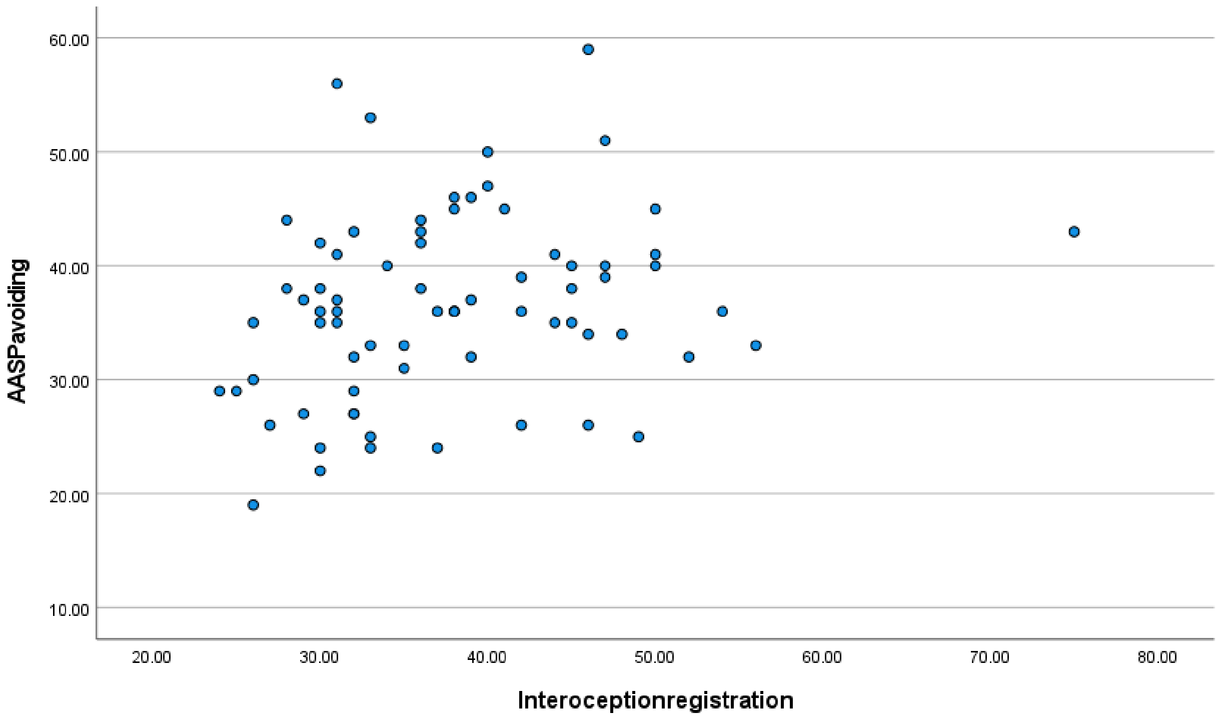

SPI seeking with A/ASP seeking

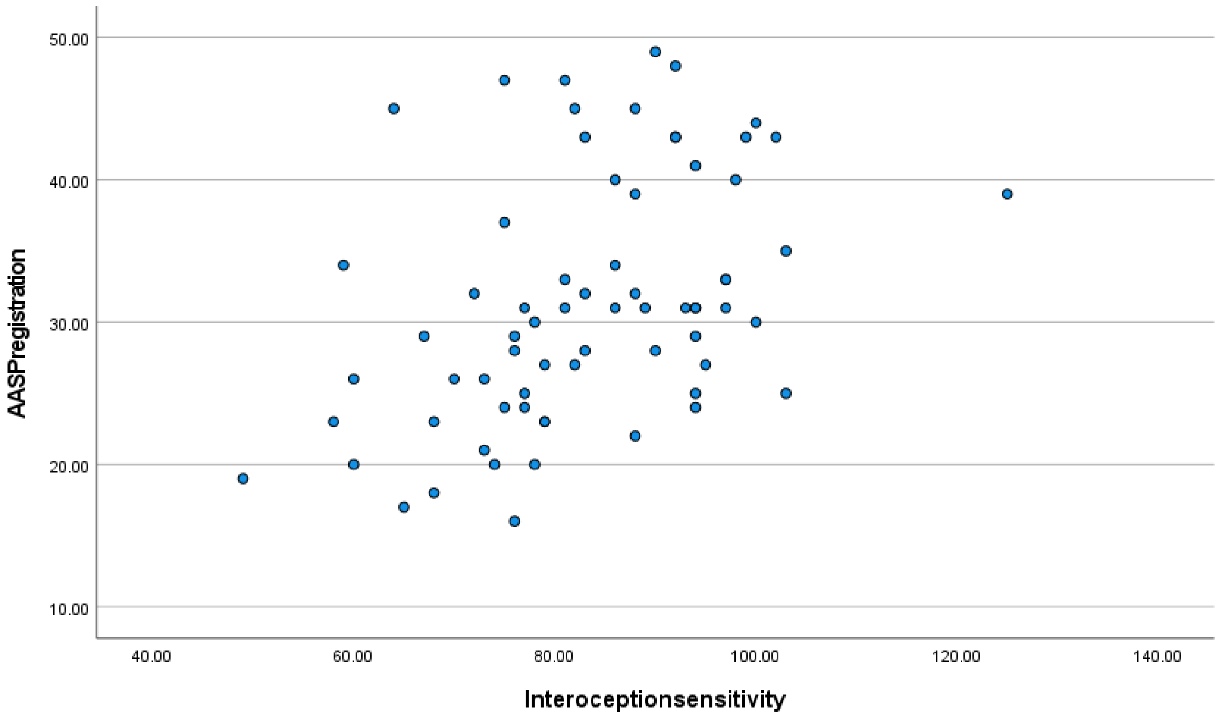


SPI sensitivity with A/ASP registration

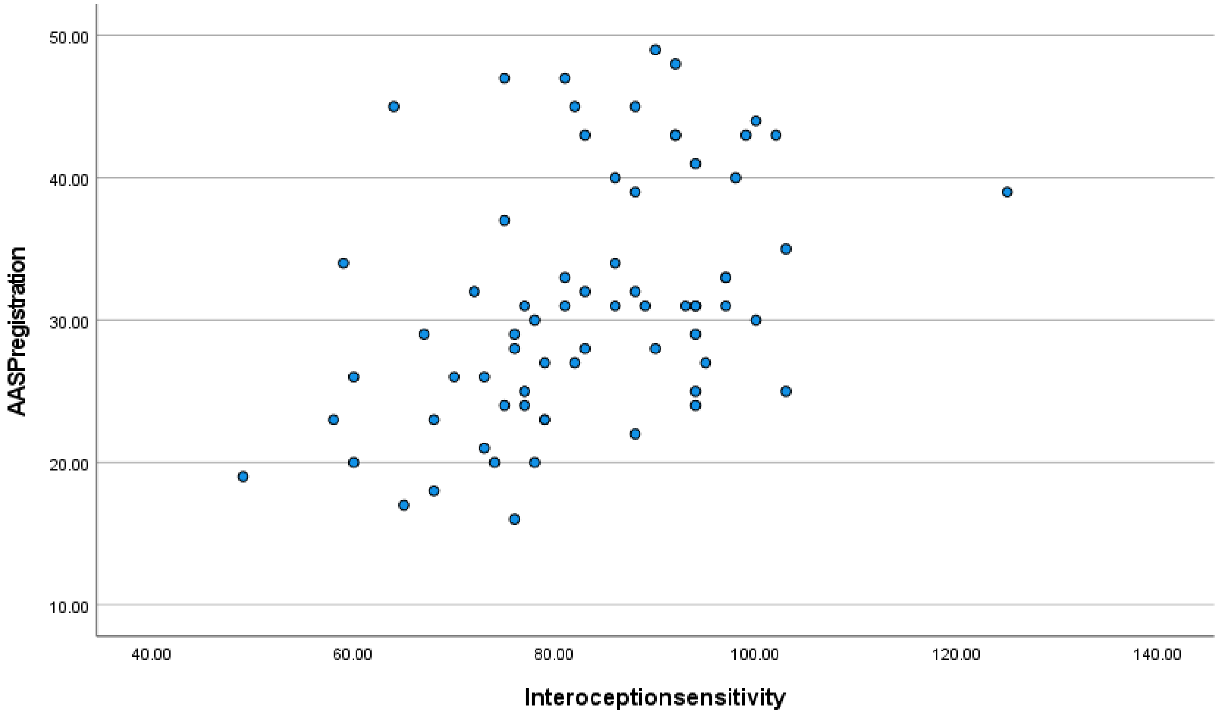

SPI sensitivity with A/ASP seeking

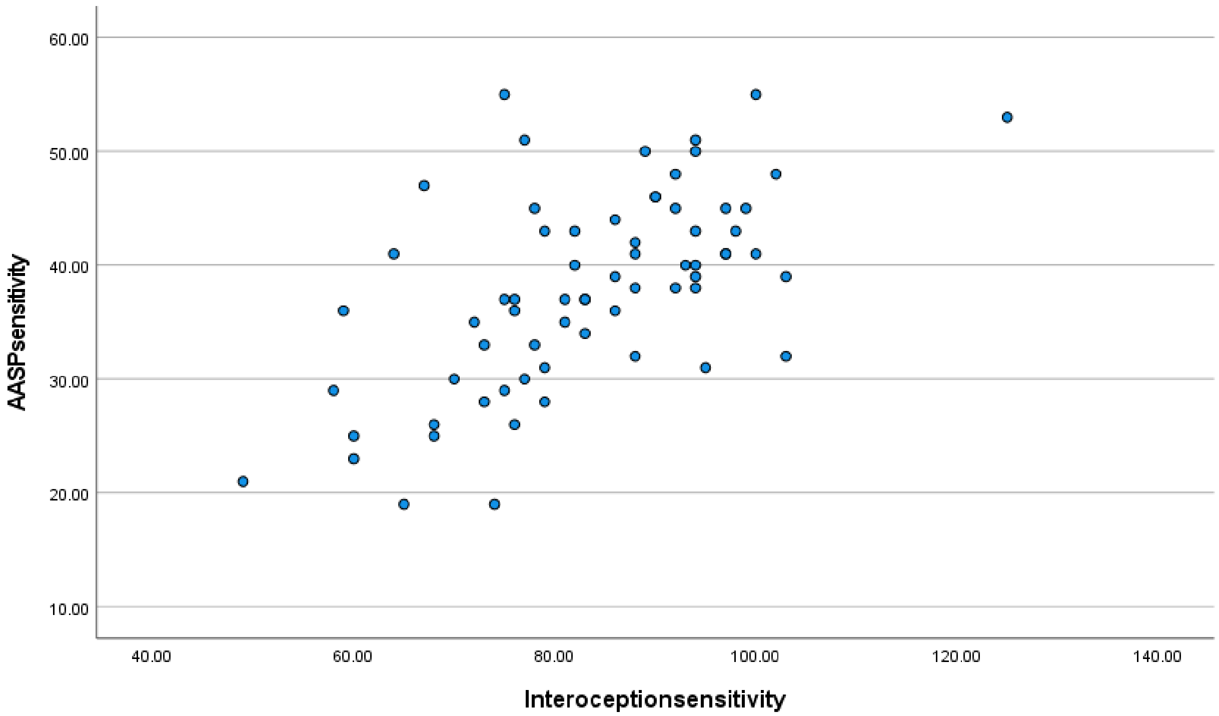


SPI sensitivity with A/ASP sensitivity

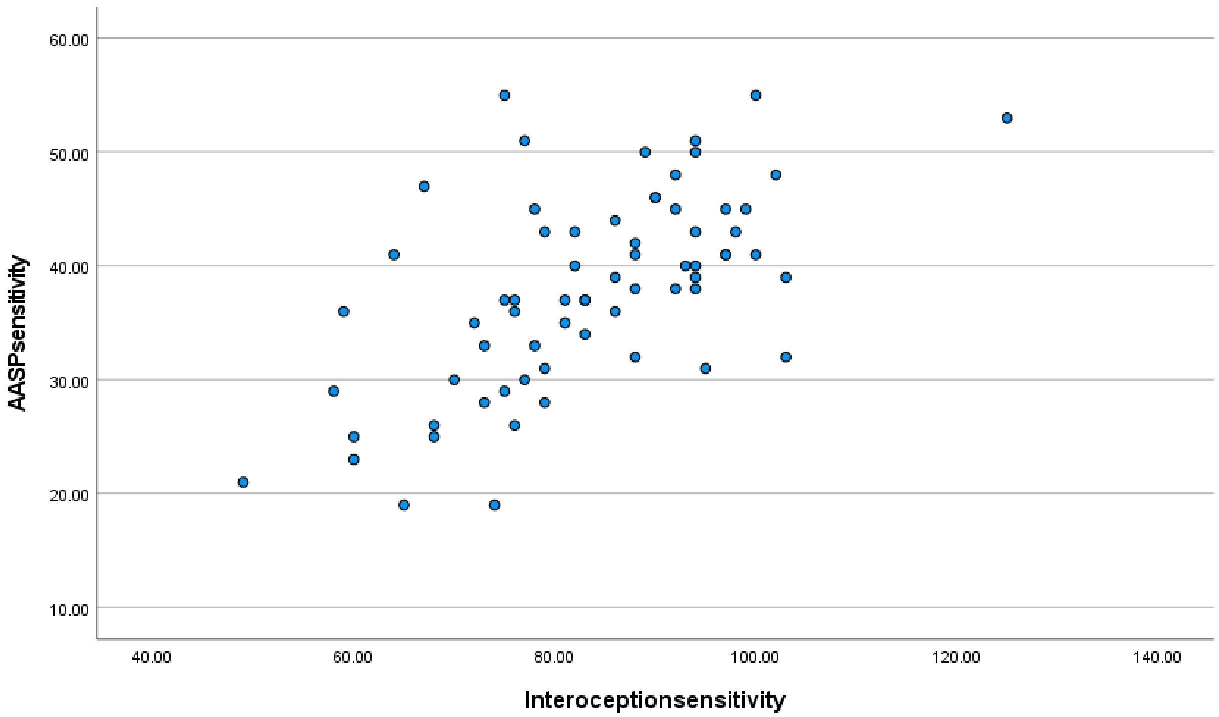

SPI sensitivity with A/ASP avoiding

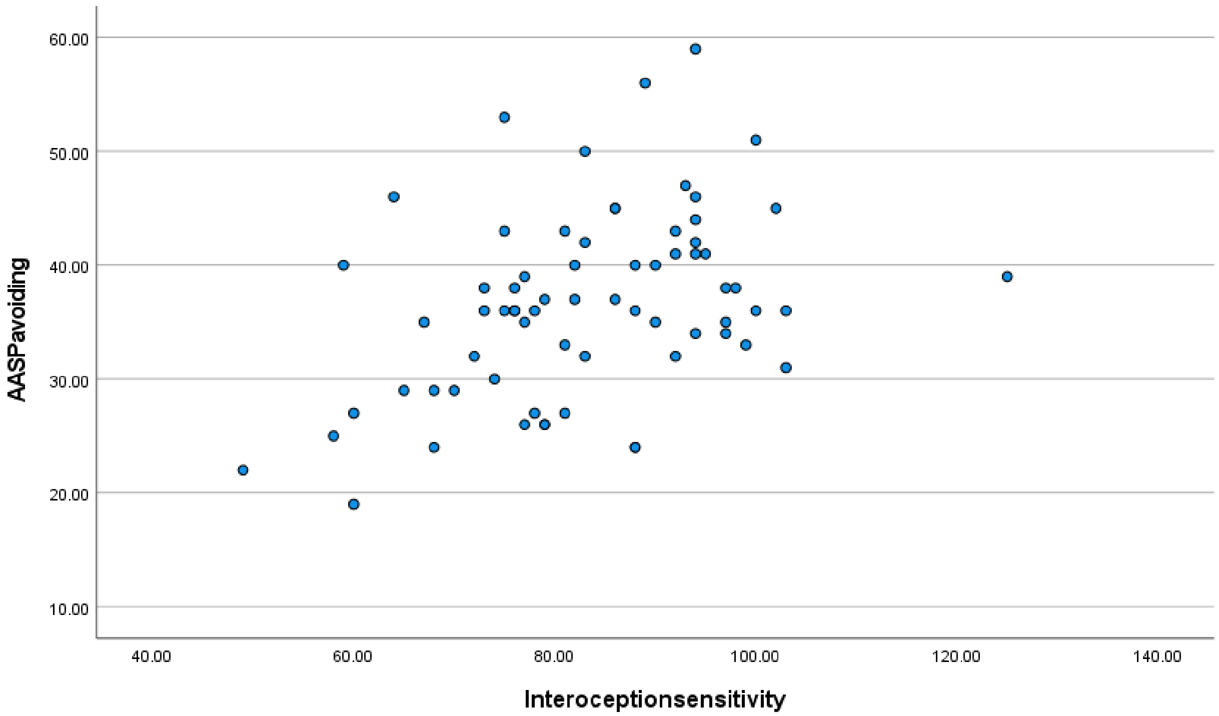


SPI avoiding with A/ASP seeking

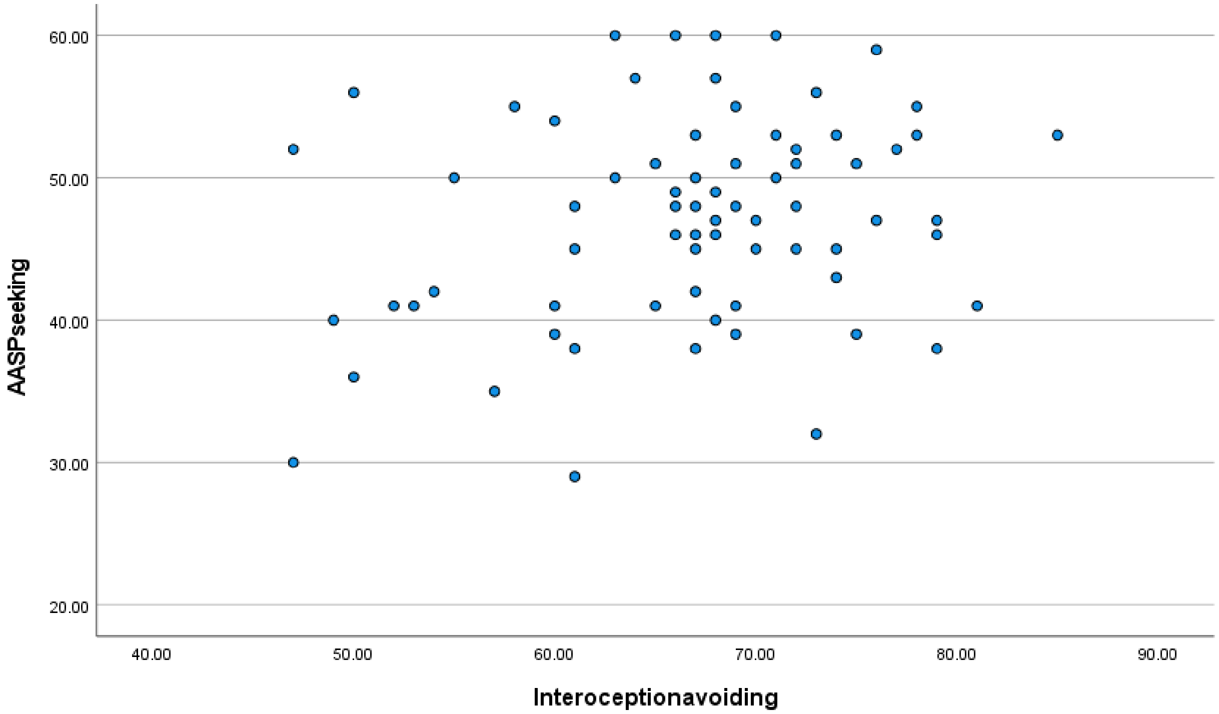

SPI avoiding with A/ASP sensitivity

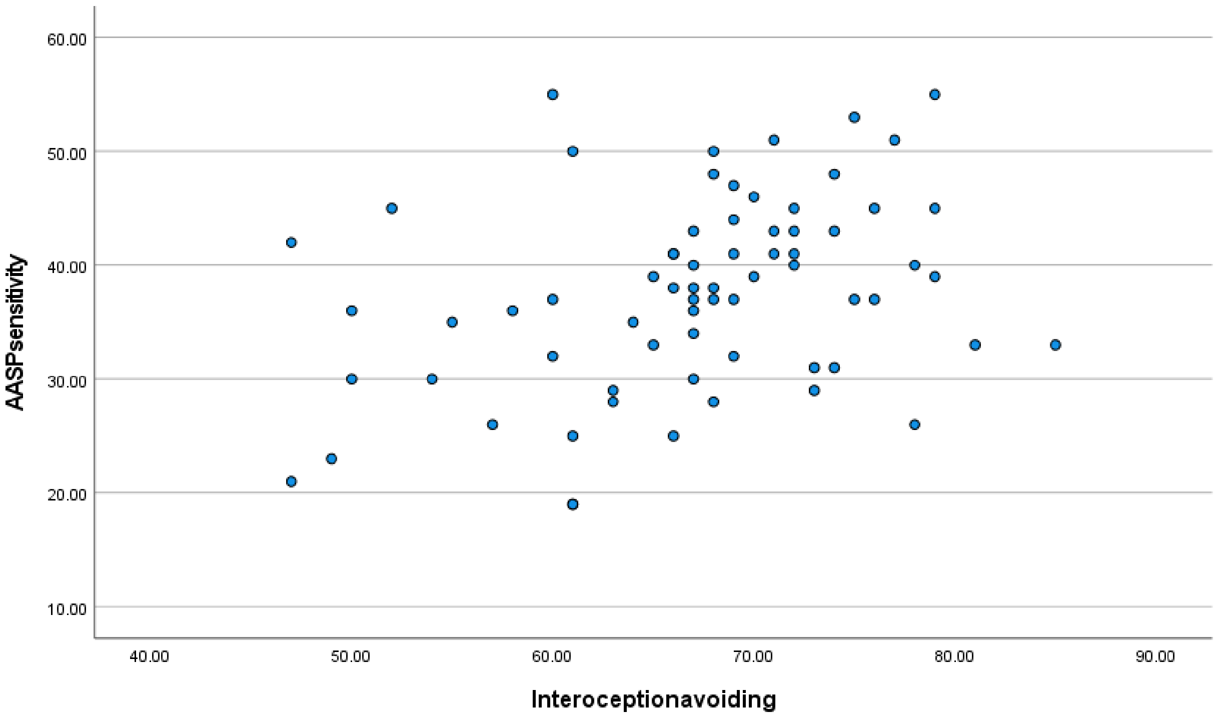


SPI avoiding with A/ASP avoiding

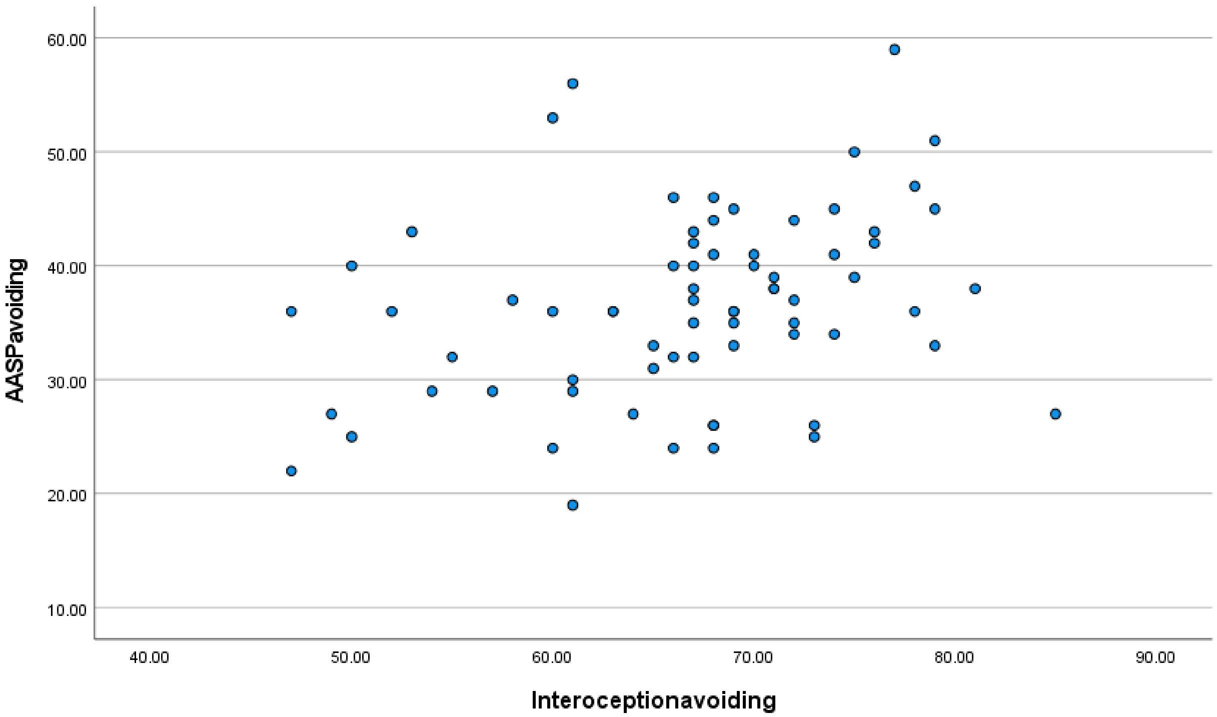

**SCATTERPLOTS for Table 4 significant correlations**

SPI avoiding with BAS

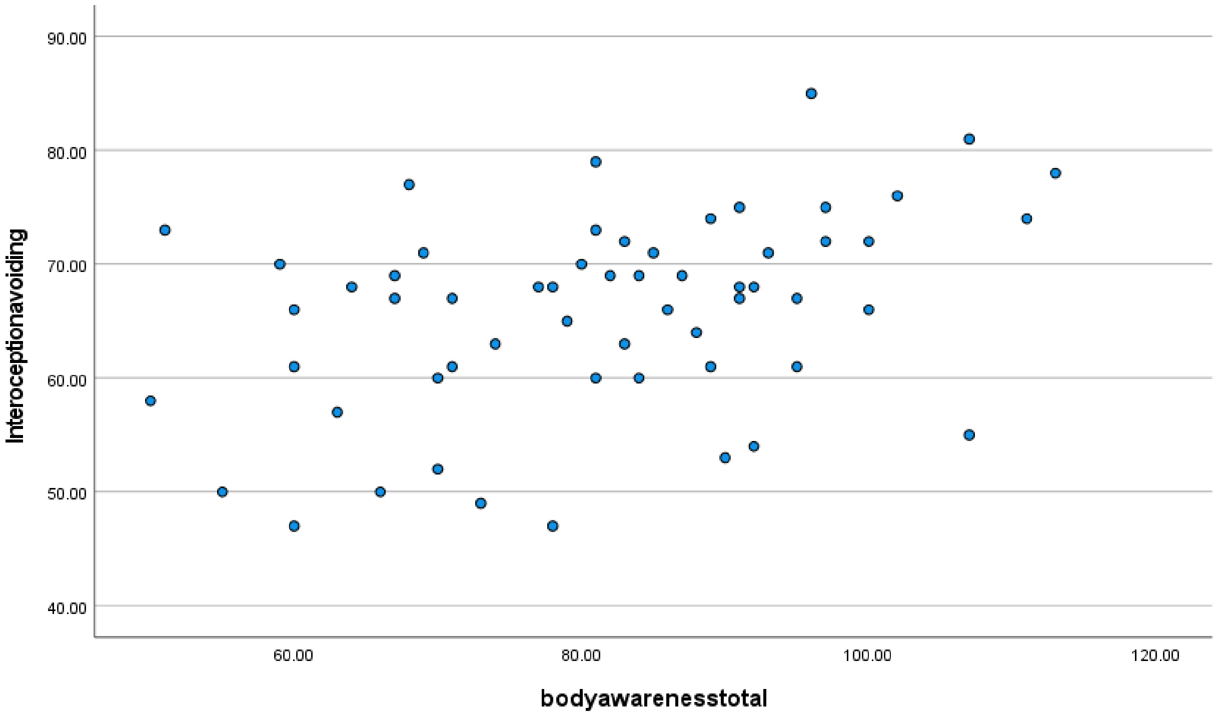


SPI sensitivity with BAS

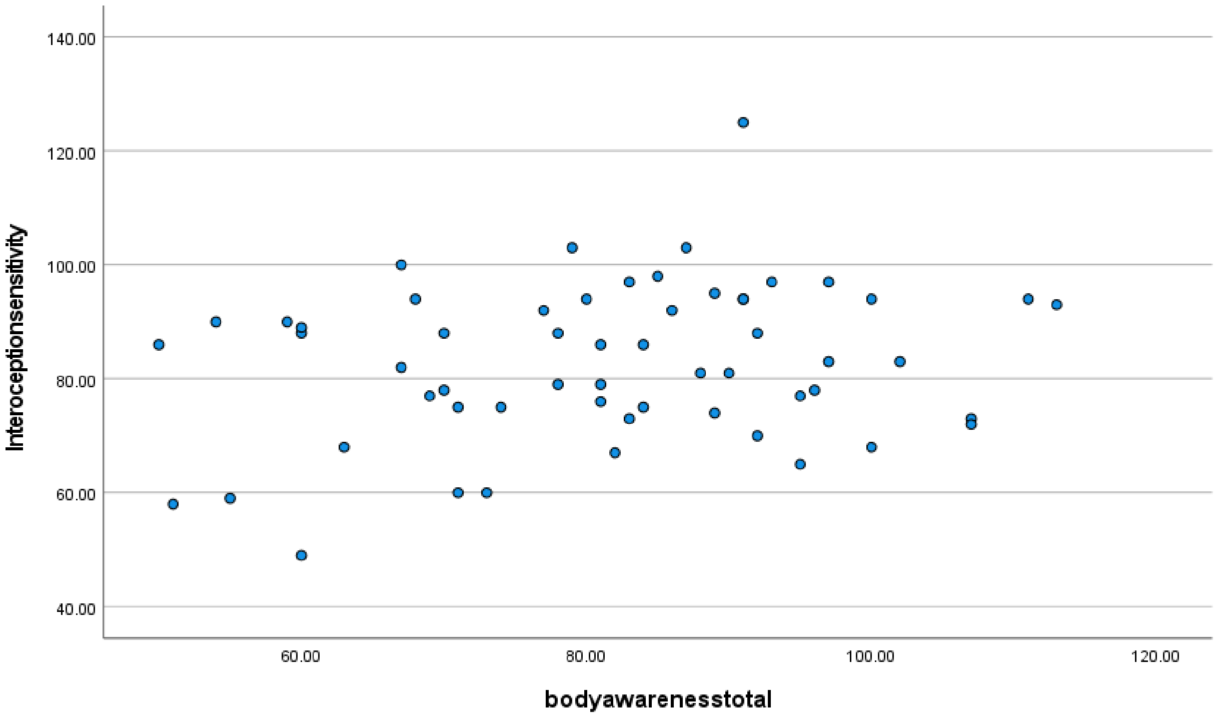

SPI sensitivity with State Anxiety

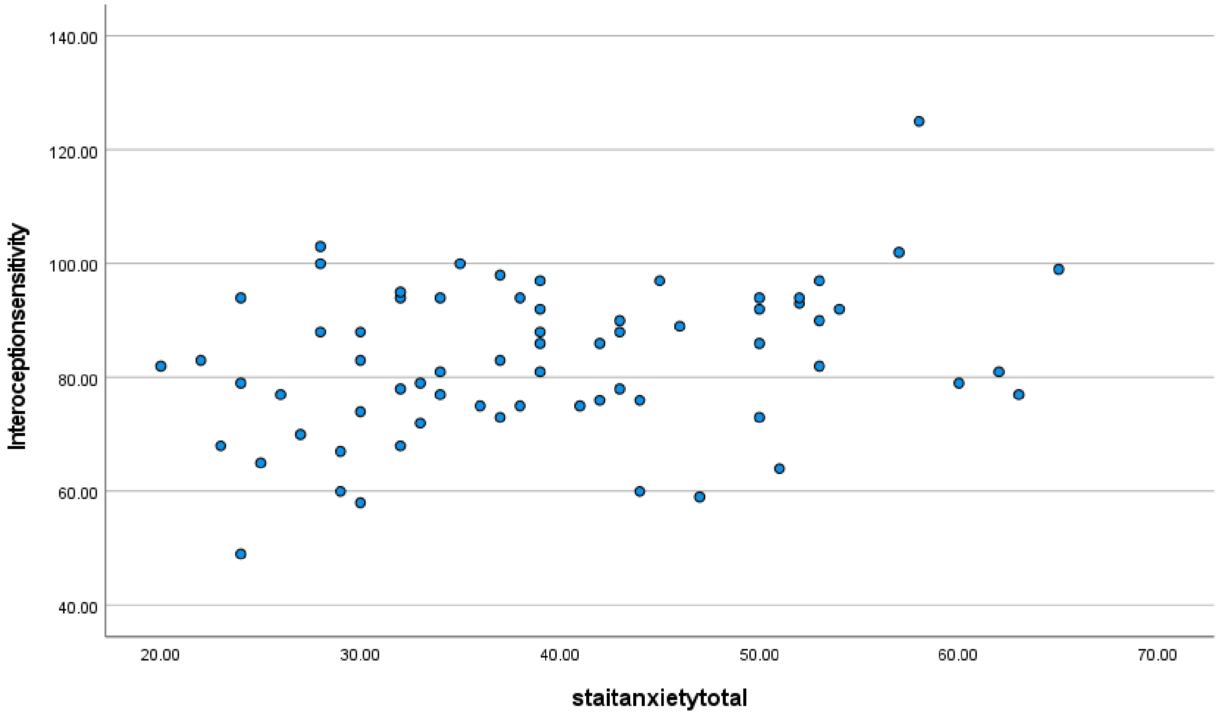


SPI sensitivity with Trait Anxiety

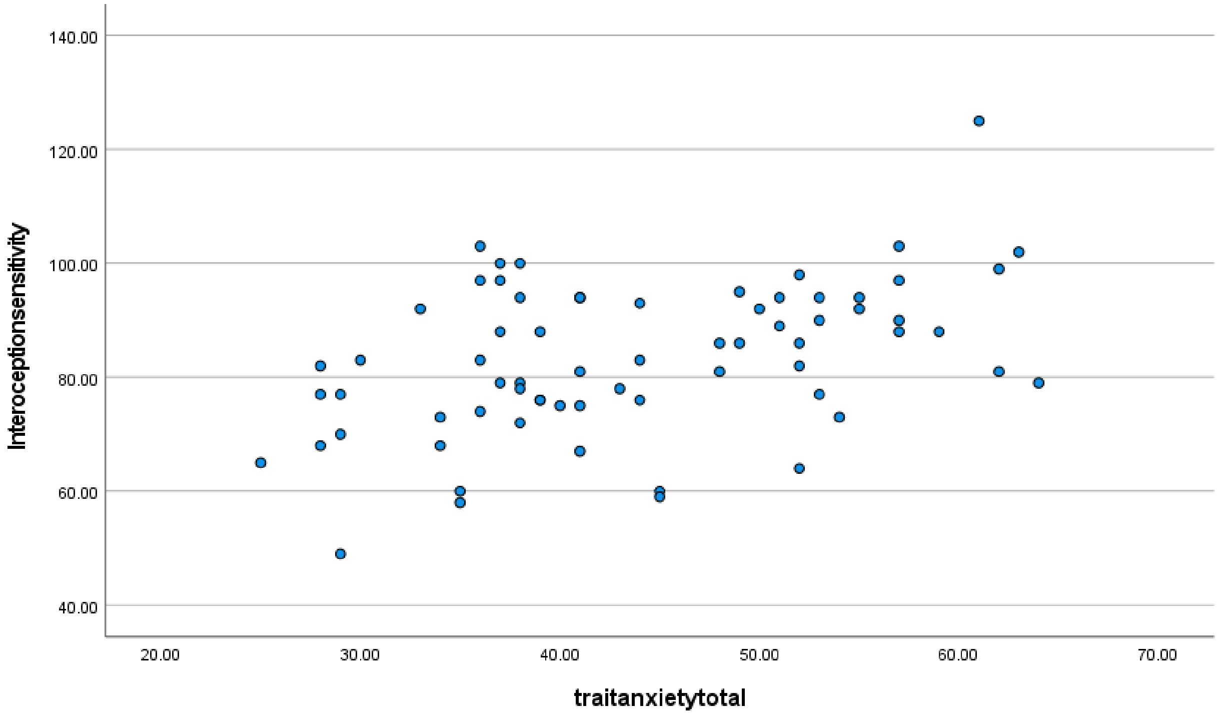

SPI registration with PAS

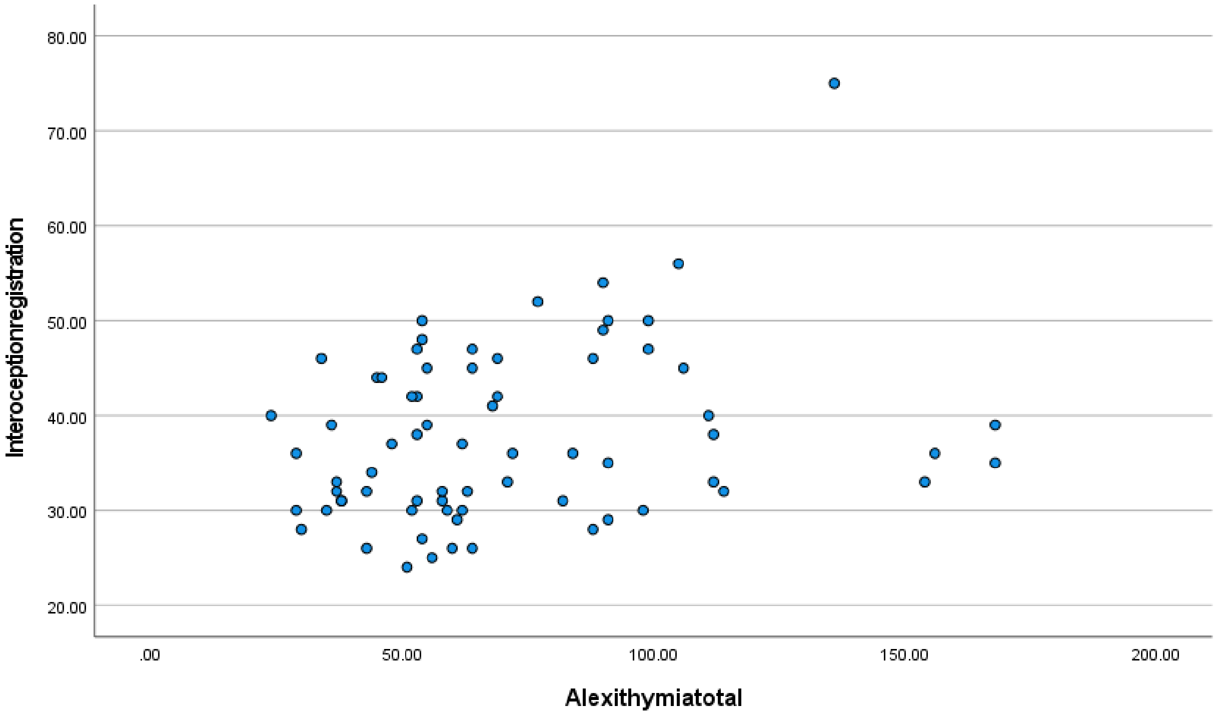


SPI registration with State Anxiety

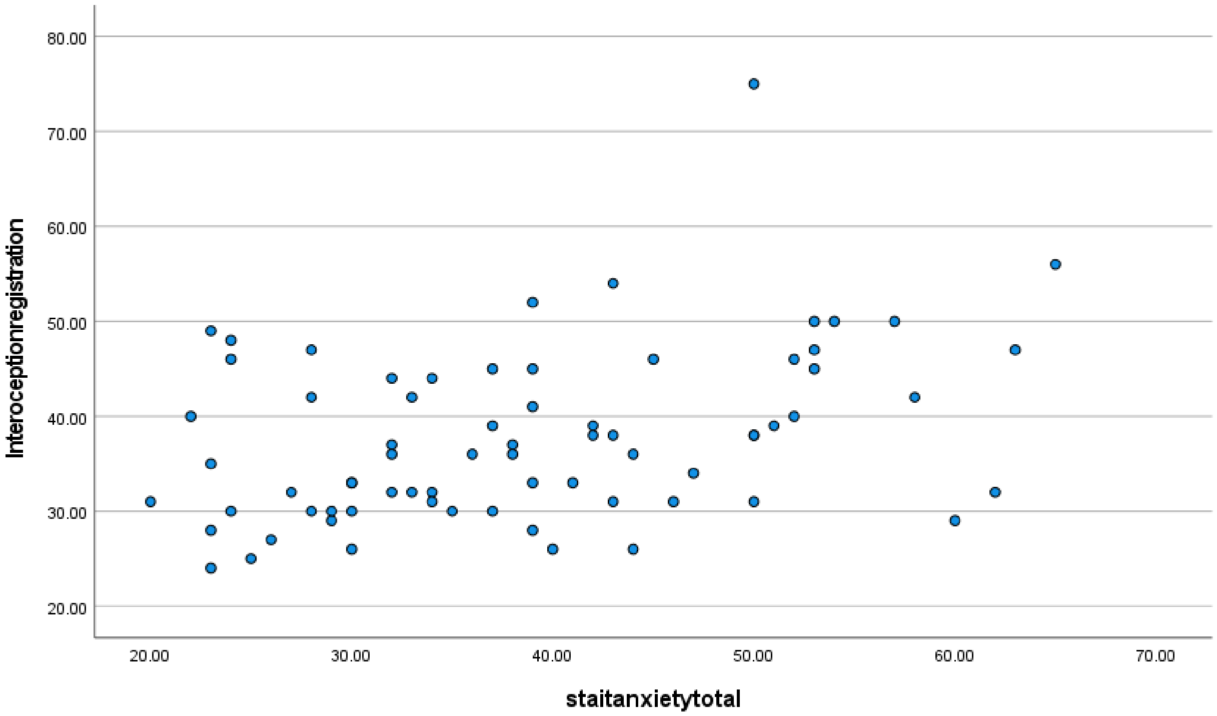

SPI registration with Trait Anxiety

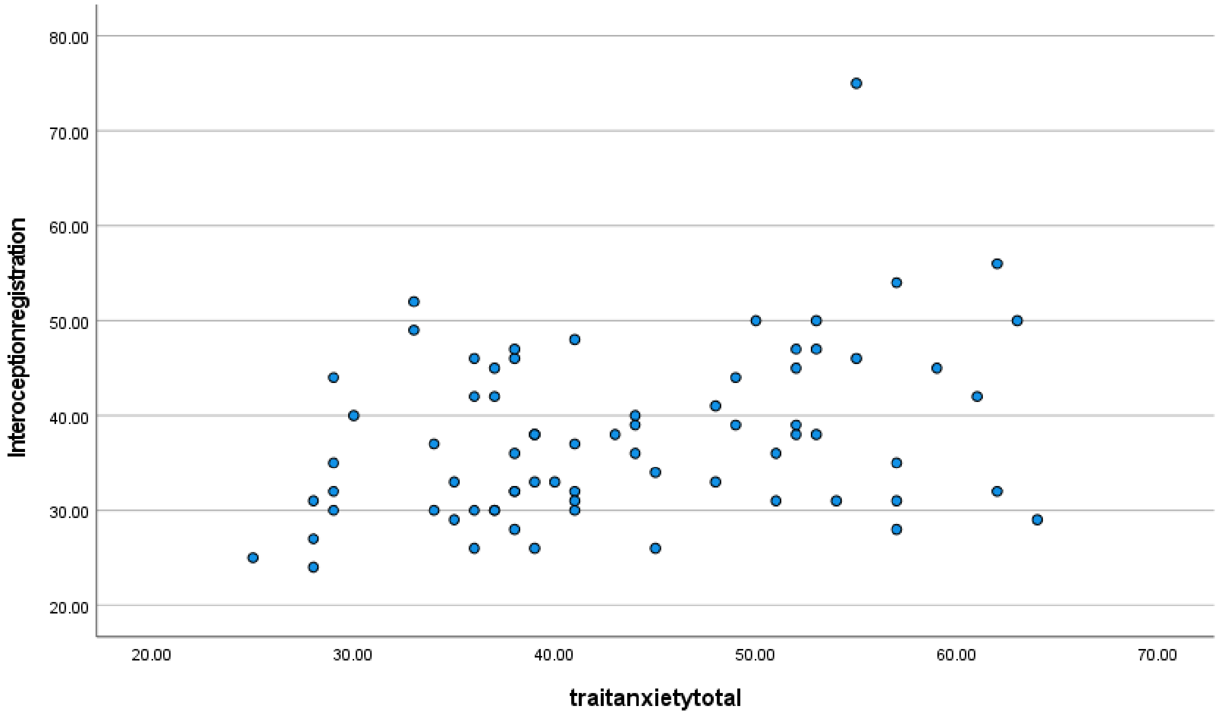


SPI seeking with BAS

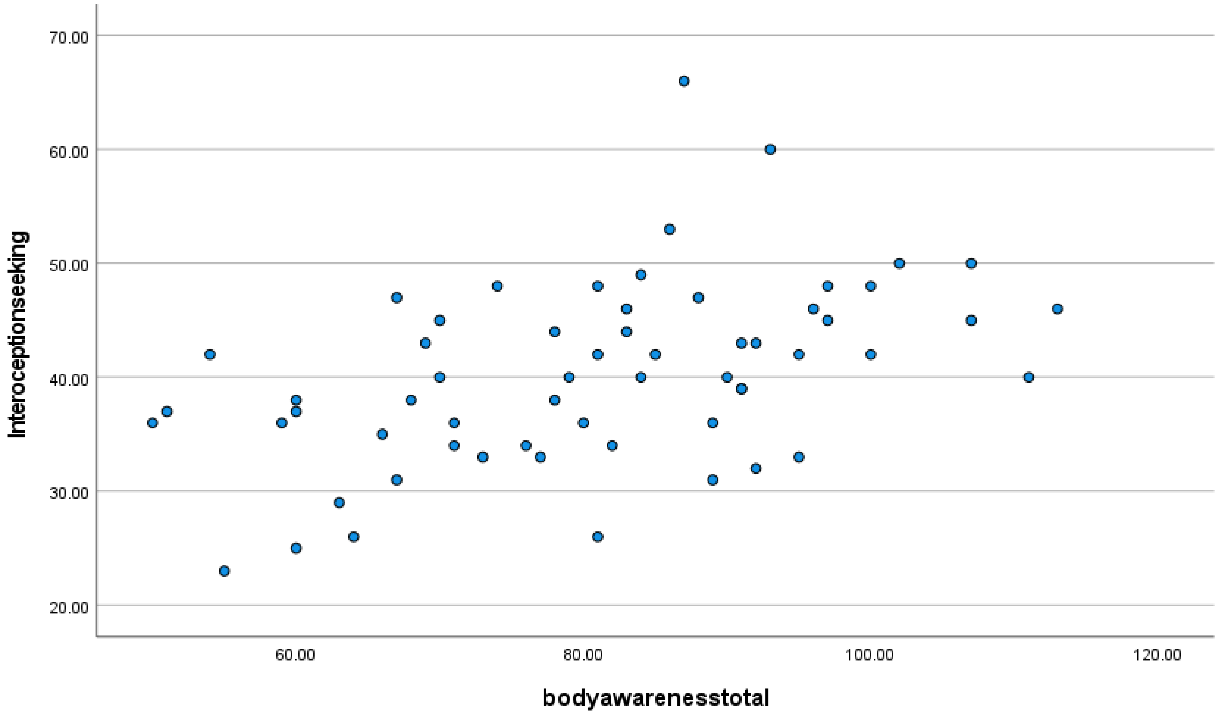

Supplement: Supplementary file 1 [file Data_Sheet_1.pdf]
